# Non-native Nematode *Ashworthius sidemi* Currently Dominates the Abomasal Parasite Community of Cervid Hosts in the Czech Republic

**DOI:** 10.3389/fvets.2022.862092

**Published:** 2022-04-28

**Authors:** Jan Magdálek, Gilles Bourgoin, Jaroslav Vadlejch

**Affiliations:** ^1^Department of Zoology and Fisheries, Faculty of Agrobiology, Food and Natural Resources, Czech University of Life Sciences Prague, Prague, Czechia; ^2^Laboratory of Veterinary Parasitology, Université de Lyon, VetAgro Sup – Campus Vétérinaire de Lyon, Marcy l'Etoile, France; ^3^Laboratoire de Biométrie et Biologie Évolutive, CNRS, UMR 5558, Université de Lyon, Université Lyon 1, Villeurbanne, France

**Keywords:** wild ruminants, gastrointestinal tract, necropsy, parasitic load, roe deer, temperature

## Abstract

*Ashworthius sidemi* is an abomasal nematode typical for Asiatic cervids such as sambar (*Rusa unicolor*) or sika deer (*Cervus nippon*). This non-native parasite was introduced into Europe *via* sika deer in the late 19th and early 20th centuries. The current dynamic spread of this parasite amongst autochthonous wild cervids occurs independently of human activities, and *A. sidemi* has a negative impact on the health of wild ruminants and may pose a threat to the conservation of endangered wild ungulates and to livestock. This invasive parasite has been previously detected in the Czech Republic, but more accurate information on *A. sidemi* is required. Only limited information is generally available on the factors influencing the spread of abomasal nematodes in wild ruminants, so more information is necessary for planning effective strategies of parasite control. We therefore conducted a survey on the abomasal nematodes in cervids in both game reserves and hunting grounds across the Czech Republic, taking into account the hosts (species, age, sex) and environmental factors (monthly average temperature). The abomasa of 104 animals belonging to five cervid species originating from various locations of the country were collected. Data on host (species, sex, and age group) and the monthly average temperature in the region were obtained for each animal. The parasitological analyses indicated that 92% of the abomasa were infected by nematodes. *Ashworthius sidemi* was the most prevalent (72%) and abundant (80% of the total recovered individuals) nematode species and was detected in all cervid species except white-tailed deer. The intensity of *A. sidemi* was highest in roe deer (*Capreolus capreolus*) and fallow deer (*Dama dama*), but *A. sidemi* abundance did not depend substantially on the host or environmental factors. In contrast, the abundance of nematodes from the subfamily Ostertagiinae was influenced by the host species and temperature. Parasitic load was significantly higher in roe deer and during the warmer periods of the survey. We also detected another non-native nematode species, *Spiculopteragia houdemeri*. The results of our study suggest that the non-native nematode *A. sidemi* is now widespread amongst cervid hosts in the Czech Republic, probably due to the high sensitivity of autochthonous cervids to *A. sidemi* infections as well as adaptation of this parasite to the current climatic conditions of this country.

## Introduction

Trichostrongylid nematodes (trichostrongylids) are globally considered to be amongst the most important parasites in ruminants. These parasites usually occur in mixed infections of several species (1, 2). The intensities of trichostrongylid infections are lower in wild ruminants than livestock, but infection may affect the health and fitness of their host under specific circumstances, with potential consequences at both the individual and population levels (3–5).

Emerging diseases associated with alien (non-native) parasites can lead to increased morbidity and mortality in native host populations (6). The introduction of alien species can take the form of an invasion if it is established and spreads on the new site. Co-invasion may occur if the parasites of the newcomer host species are transmitted and established in the populations of the native hosts. The virulence of alien parasites is also often higher in native hosts than in the original (alien) host (7). Introduction of non-native trichostrongylid nematodes thus can pose a threat to endangered wild ruminants and hinder reintroduction efforts (8, 9).

Several studies on cervids have found that abomasal nematodes were the most prevalent trichostrongylids of the gastrointestinal (GI) tract, with the subfamily Ostertagiinae (ostertagids) repeatedly reported as the dominant GI parasites of wild cervids in Europe and North America (10–12). The diversity of communities of parasitic nematodes, however, can vary over time and space (13). An association of a parasite with a new host is allowed if they encounter one another and are biologically compatible (14). Biocompatible parasites can range from specialists to generalists (capable of infecting a limited number of hosts and a wide range of hosts, respectively). For example, the majority of the abomasal nematodes of ruminants are considered generalists (15, 16). Barriers preventing encounters can be penetrated under specific circumstances, e.g., due to ecological perturbations or human activity (17, 18). Anthropogenic translocations of domestic sheep and goats have historically caused the colonization of Nearctic cervids by *Haemonchus contortus* (subfamily Haemonchiinae), which is currently a dominant parasite of white-tailed deer (*Odocoileus virginianus*) in North America (12).

The non-native abomasal nematode *Ashworthius sidemi* (subfamily Haemonchiinae) has more recently emerged amongst European ruminants This parasite occurs primarily in Asiatic cervids. It has been described in sika deer (*Cervus nippon hortulorum*) from the far east of Russia (19) but occurs naturally in sambar (*Rusa unicolor*) from northern Vietnam (20). *Ashworthius sidemi* in European wild ruminants has been frequently reported since the last decade of the 20th century, but this blood-sucking nematode was probably introduced into Europe *via* sika deer (*Cervus nippon*) a century earlier. This host has been introduced into several European countries to diversify the species spectrum of game. Imported sika deer belong mainly to the Japanese (*Cervus nippon nippon*) and eastern Russian subspecies (*Cervus nippon hortulorum*), but various subspecies from different areas have been mixed and intercrossed during its acclimatization in Europe (21). Animals were initially kept in game reserves and deer parks until World War II. Many fences were later damaged, and escaped individuals gave rise to the current free populations (21, 22). Studies from countries where sika deer were also introduced, such as France or Ukraine, indicated that *A. sidemi* became a part of the parasitic fauna of other cervid species such as the fallow deer (*Dama dama*), red deer (*Cervus elaphus*), and roe deer (*Capreolus capreolus*) (23–25). The migratory behavior of some cervid species probably allowed the spread of this alien parasite into new areas and the colonization of further hosts, including endangered species. For example, *A. sidemi* was not detected in Poland until the late 1990s. This nematode subsequently became a major pathogen of populations of the European bison from two distant areas during a relatively short period. Infection of this host has been associated with abomasitis (26) and with a decrease in the number of red blood cells (27). Migrating red deer was considered to be the source of infection in both cases (28, 29). Populations of endangered Tatra chamois (*Rupicapra rupicapra tatrica*) currently face a similar threat, because *A. sidemi* was found in Alpine chamois (*Rupicapra rupicapra*) and the sympatric red deer (8). More recently, increases in *A. sidemi* prevalence and infection intensity have been reported in locations where the parasite occurs (30) and has dynamically spread to a region where it had not been previously recorded (31). Further unintended introductions may also occur with the translocations of wild ruminants (32).

The population dynamics of parasites and their spread amongst sylvatic hosts are affected by both environmental and biological factors (18). Trichostrongylids develop directly with free-living stages, so their dynamics depend strongly on seasonal changes in temperature and humidity (33). Larval activity and development are inhibited or completely stopped at low temperatures. Conversely, energy reserves are depleted at long-term high temperatures due to hyperactivity and larval deaths (34). Sufficient moisture is necessary for the larvae to hatch and disperse on the pasture (35). The level of parasitic load may also be affected by the age and sex of the host due to differences in behavior and immune status (36).

These factors are essential for our understanding of the biology and epidemiology of these parasites, but only limited data for cervid hosts are available (37, 38). Likewise, information about the communities of abomasal nematodes in wild ruminants in the Czech Republic (CR) is still scarce. No comprehensive data on the prevalence and abundance of this invasive parasite, and the ecological factors influencing them, have yet been published, even though *A. sidemi* was detected in the CR in the last century (39).

We aimed to fill this knowledge gap using abomasa collected from wild ruminants from both game reserves and hunting grounds in various regions of the CR to: (i) identify the species richness of abomasal nematodes, with an emphasis on *A. sidemi*, and (ii) evaluate the effects of host and environmental factors on the abundance of abomasal nematodes.

## Materials and Methods

### Study Areas and Animals

**Figure 1:**
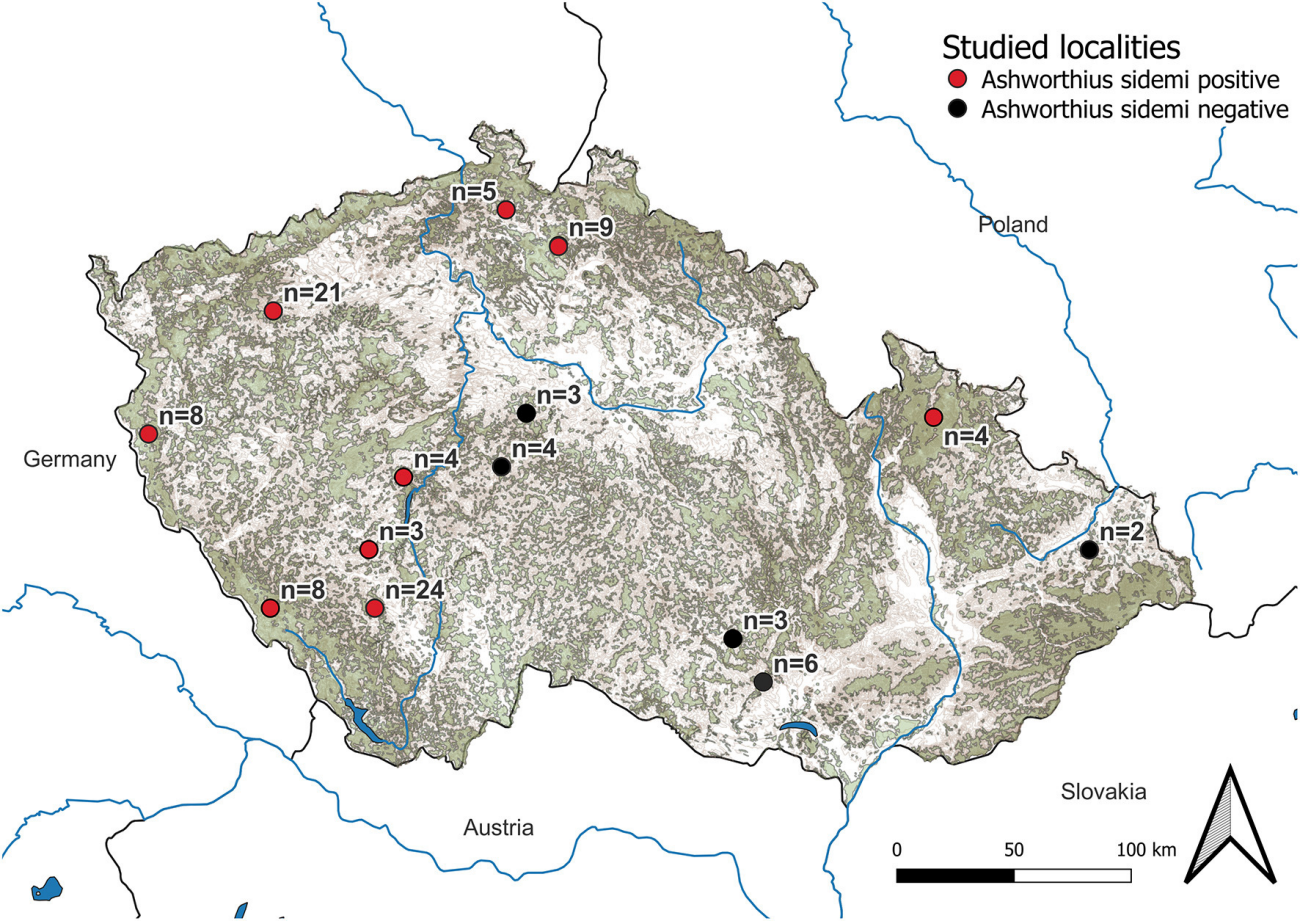
Map of localities investigated. Forest covers are represented in khaki green color and mountains are presented in brown color. The number of examined cervid hosts at each locality is listed as well.

The research was conducted in both game reserves and hunting grounds across the CR (central Europe) (Figure 1). A European continental climate with warm, dry summers and relatively cold winters is typical for the CR (40). The average temperature in each locality at the time of culling was obtained from the Czech Hydrometeorological Institute. The following three groups were formed based on temperature: (i) cold (<0°C, *n* = 31), (ii) mild (0–5°C, *n* = 60), and (iii) warm (5–16°C, *n* = 8).

GI tracts were collected by state-owned forestry companies, owners of game reserves, or individual gamekeepers from wild ruminants culled in accordance with the National Hunting Regulations during the hunting seasons of 2016–2019. An additional abomasum was opportunistically collected from a dead moose following collision with a vehicle.

A total of 104 abomasa was collected and necropsied for parasites. The animals included both species common in the CR, such as roe deer (*n* = 40), fallow deer (*n* = 31), red deer (*n* = 20), and sika deer (*n* = 10), and less-common species, one moose (*Alces alces*) and two white-tailed deer (*Odocoileus virginianus*). The latter species naturally occurs in the Nearctic region and was repeatedly introduced into the CR. A total of 24 (77%) fallow deer involved in this study were culled at a game reserve with high animal density (10 animals per hectare). The animals were annually dewormed at the beginning of the year in January 2018 and 2019.

The ages of the animals were estimated based on tooth wear (both sexes) and the development of antlers in males (41, 42). The animals were then assigned to three age groups: (i) fawns younger than 1 year (*n* = 26), (ii) yearlings and young individuals (*n* = 26), and (iii) adults older than 3 years (*n* = 47).

### Necropsies

The GI tracts were transported to the laboratory as soon as possible after evisceration. Abomasa were subjected to a complete parasitological necropsy, including the collection of larval stages in tissues (43). Ligated abomasa were separated from the rest of the GI tract and then opened longitudinally along the greater curvature. Organ contents together with mucosal washings were rinsed into separate buckets and then poured through 200 and 150-μm mesh sieves. Abomasal mucous membranes were then immersed in a saline solution and left overnight at room temperature to release arrested larval stages. The fluid obtained was poured through a 38-μm mesh sieve to separate released larvae from the debris (44). All recovered nematodes were fixed in 70% ethanol until processed. The nematodes were briefly immersed in a phenol-alcohol solution (80 parts of melted phenol crystals and 20 parts of a 96% ethanol solution) before examination to clarify the visibility of morphological characters.

Nematodes belonging to the subfamily Haemonchiinae were identified based on the morphology of the neodont and deirids, the morphology and length of spicules, and the structure of the copulatory bursa (24, 45). All males were examined if fewer than 100 individuals were recovered; at least ten percent of the males from the subfamily Haemonchiinae were examined in all cases. All recovered male ostertagids were identified based on the structure of the copulatory bursa and the structure and length of spicules (46) taking polymorphism into account (47). Female ostertagids were pooled into one group, because their morphological identification is both extremely difficult and time-consuming. Nematode larvae were likewise identified only as fourth (L4) stage. The nematodes were studied using an Olympus BX51 microscope with a Promicam 3-3CP Sony Pregius camera running QuickPHOTO MICRO 3.0 software.

### Data Analysis

We used three measures to describe parasitic loads: prevalence, intensity, abundance, and species richness. The 95% confidence intervals (CI) for the quantitative descriptors of parasitic loads were calculated. Prevalence was calculated as the proportion of individuals of a particular host species infected with a particular nematode species from all hosts examined. Intensity was calculated as the number of nematodes recovered from an infected host. Abundance was calculated as the number of nematodes in the hosts examined regardless of whether they were infected. Species richness was expressed as the number of nematode species identified in a particular host species. Species richness was compared between species, sexes, age groups, and temperature categories (described in Study areas and animals) using the non-parametric Kruskal-Wallis test. Significant results were visualized using the ggplot2 R package (48). The effects of environmental (temperature, expressed as a continuous variable) and host (species, sex, and age) factors on parasite abundance were estimated using multiple regression on the abundance of *A. sidemi* and pooled ostertagids (log-transformed to achieve a normal distribution). Two white-tailed deer and one moose were excluded from the analysis of abundance because of their low numbers. We selected the model with the lowest Akaike information criterion corrected for small sample size (AICc) and retained the simplest model (49) when two or more competing models had a ΔAICc < 2. The normality of the residuals was assessed using the Lilliefors (Kolmogorov-Smirnov) test. All calculations and statistical tests were performed using R 4.0 (50).

## Results

Abomasal nematodes were recovered from 92% [95% CI = 85–96] of all cervids; the prevalences of the nematode species identified in this survey are presented in Table 1. The median intensity was 102 individuals [95% CI = 69–176]. The total number of nematodes detected in the abomasa of the cervids was almost 27,000. Histotrophic (tissue dwelling) larvae were recovered in low numbers (from 1 to 26 individuals) from 7% [95% CI = 3–13] of the mucous membranes.

**Table 1:** Prevalence of abomasal nematodes in the main cervids culled between 2016 and 2019. Moose (single case) and white-tailed deer (*n* = 2) are not included in the table due to the low number of individuals examined.

	**Host species**			
	**Roe deer (*****n*** **=** **40)**	**Red deer (*****n*** **=** **20)**	**Fallow deer (*****n*** **=** **31)**	**Sika deer (*****n*** **=** **10)**
**Nematode species**		**Prevalence % (CI)**		
Overall nematodes	98 (85–99)	95 (73–99)	83 (66–94)	99 (54–99)
*Ashworthius sidemi*	75 (60–80)	65 (43–82)	74 (57–87)	80 (48–95)
*Haemonchus contortus*	12 (5–26)	–	–	–
*Ostertagia leptospicularis/kolchida*	73 (57–84)	40 (22–61)	48 (32–65)	10 (0.2–45)
*Spiculopteragia spiculoptera/mathevossiani*	45 (35–60)	40 (22–61)	3 (0.8–17)	30 (10–60)
*Spiculopteragia asymmetrica*	10 (3–20)	5 (0.1–25)	39 (24–56)	20 (5–52)
*Spiculopteragia houdemeri*	20 (10–35)	5 (0.1–25)	3 (0.8–17)	40 (17–69)

*CI, upper and lower limit of 95% confidence interval based on the binomial distribution*.

**Figure 2:**
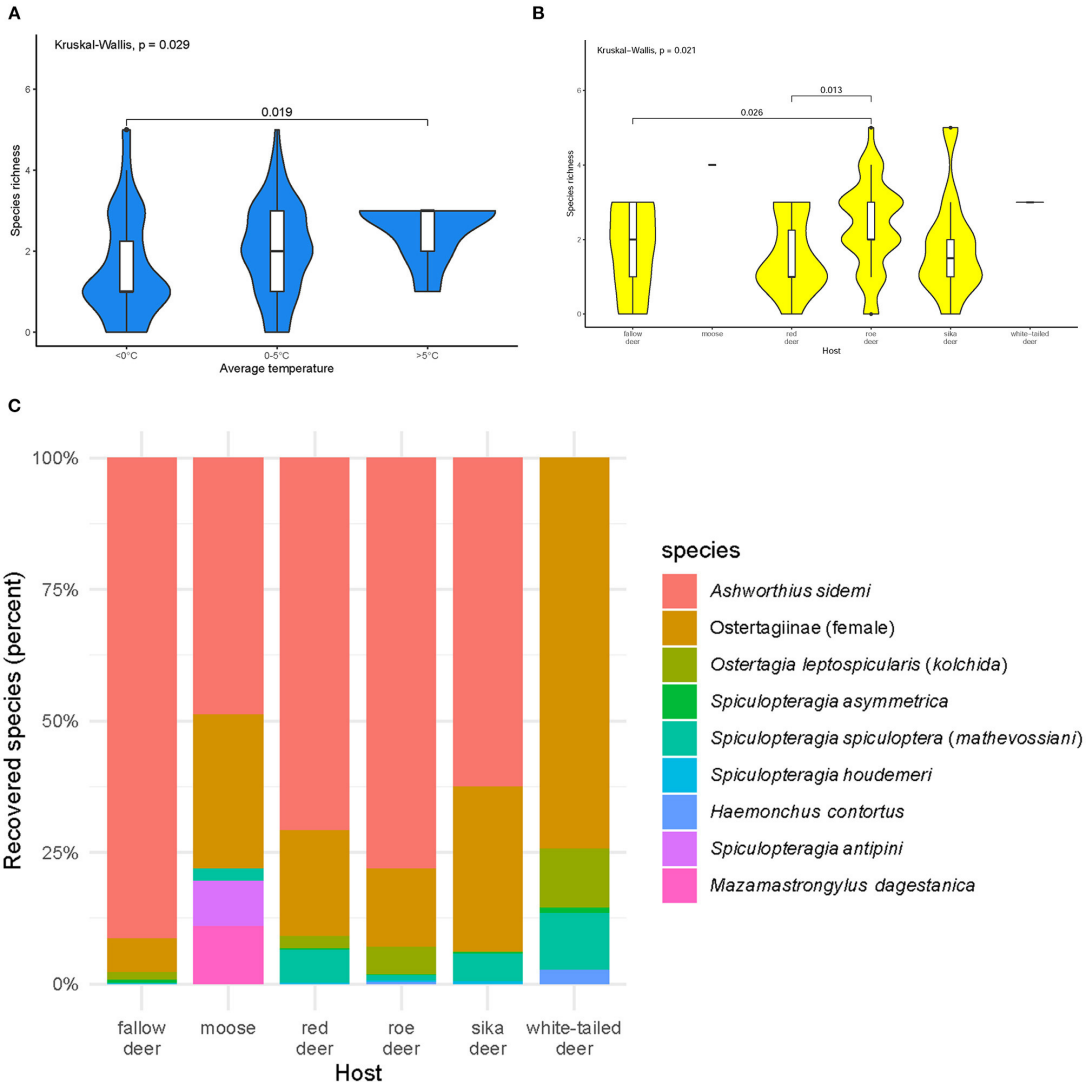
Species richness of abomasal nematodes **(A)** in periods with different average temperatures **(B)** in examined host species, and **(C)** community structure of abomasal nematodes identified in the cervid hosts during necropsies: fallow deer (*n* = 31), moose (*n* = 1), red deer (*n* = 20), roe deer (*n* = 40), sika deer (*n* = 10), and white-tailed deer (*n* = 2). Females from the subfamily Ostertagiinae were merged into one group. Black dots symbolize outliers. Host species are sorted alphabetically.

Most of the abomasa were infected with one (*n* = 26%), two (*n* = 26%), or three (*n* = 29%) nematode species. Species richness varied during the year (*p* = 0.04). Animals culled during the warmer times of the year (5–16°C) were infected with more species than those culled in winter (Figure 2A). Species richness was highest in roe deer (Figure 2B).

**Figure 3:**
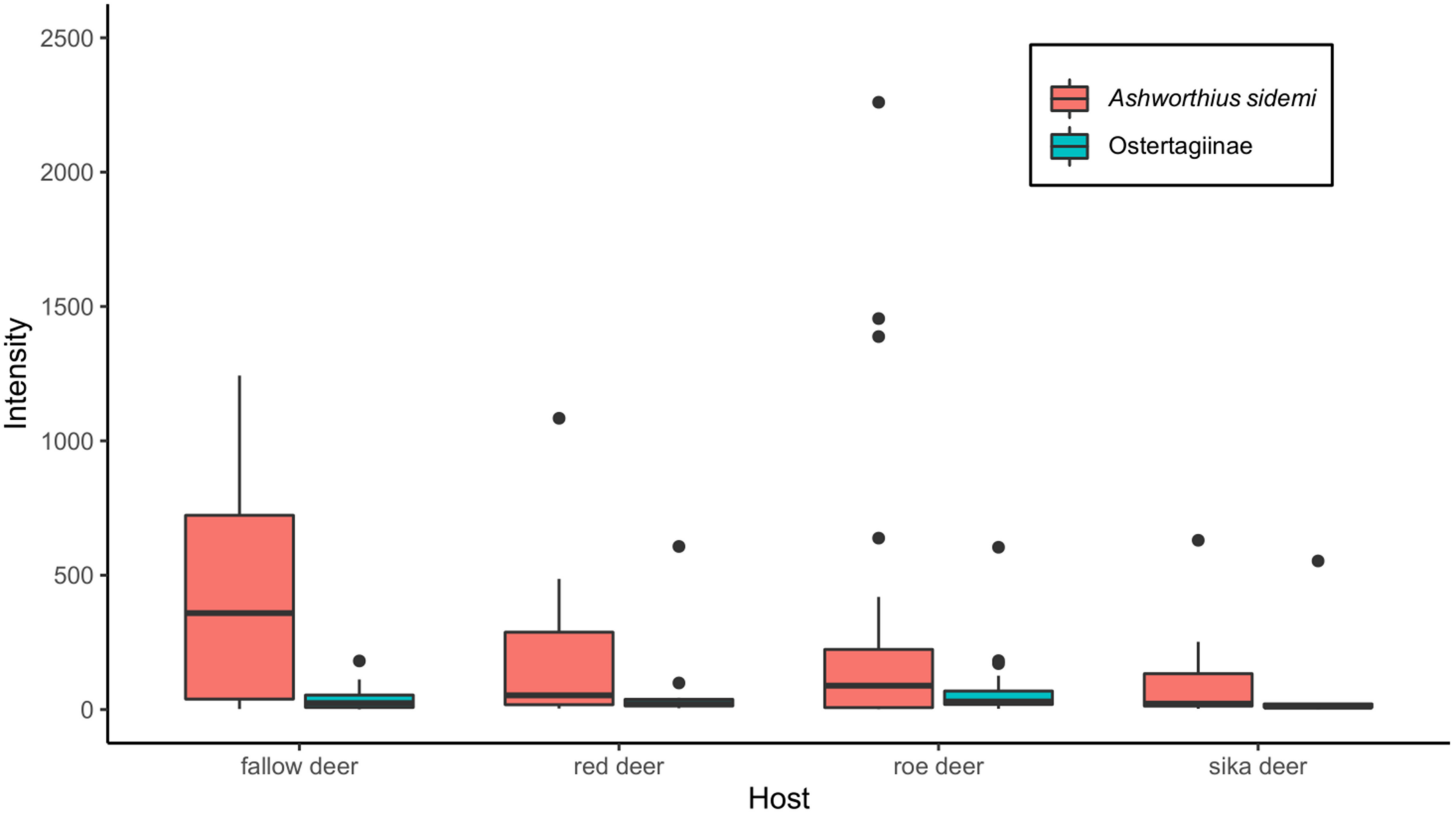
Median intensity of both *Ashworhius sidemi* and nematodes from the subfamily Ostertagiinae in the evaluated cervid host species presented as boxplot. Black dots symbolize outliers.

*Ashworthius sidemi* was detected in 72% [95% CI = 63– 80] of the animals and was the most abundant nematode (*n* = 21,646; 80% of all nematodes). This parasite was a dominant component of the community of abomasal nematodes in most of the cervid species (Figure 2C) and occurred in 9 of 14 localities (Figure 1). The median intensity of *A. sidemi* infections in cervids was 86 [95% CI = 40–197], with a maximal intensity of infection (*n* = 2,260) in a three-year-old female roe deer. Intensities of *A. sidemi* detected in the evaluated cervid hosts are shown in Figure 3.

The infrapopulation of *A. sidemi* consisted predominantly of adults. Juveniles occurred in 16% [95% CI = 10–25] of the abomasa and formed a minor part of the infrapopulation. The numbers of these early adults varied from 1 to 81. Based on the selected model (Supplementary Table 1A), the abundance of *A. sidemi* did not significantly depend on host or environmental factors.

Five roe deer and one white-tailed deer were infected with *H. contortus* (6% prevalence [95% CI = 2–13] of all cervids). Co-infection with *A. sidemi* and *H. contortus* was not observed in any of the abomasa.

Polymorphic ostertagids were the most frequent (77% prevalence [95% CI = 67–84]) abomasal nematodes recovered from the culled cervids. The intensity of infection with these nematodes ranged from 1 to 607 adults. The median intensity was 26 (95% CI = 20–42). For intensity of nematodes from the subfamily Ostertagiinae detected in the evaluated cervid hosts (see Figure 3). *Ostertagia leptospicularis* and *O. kolchida* were the most frequently identified ostertagids (52% prevalence [95% CI = 43–63]), followed by *Spiculopteragia spiculoptera* and *S. mathevossiani* (32% prevalence [95% CI = 23–42]). These nematodes were common in roe deer and red deer, and *Spiculopteragia asymmetrica* (19% prevalence [95% CI = 12–28]) was recovered mainly from fallow deer and sika deer (Table 1). *Spiculopteragia houdemeri* (13% prevalence [95% CI = 8–21]) was detected in red deer, roe deer, fallow deer, and sika deer. Minor male morphotypes of *S. asymmetrica* and *S. houdemeri*: *Spiculoptertagia quadrispiculata* and *Spiculopteragia andreevae*, respectively, were not detected during this survey.

**Figure 4:**
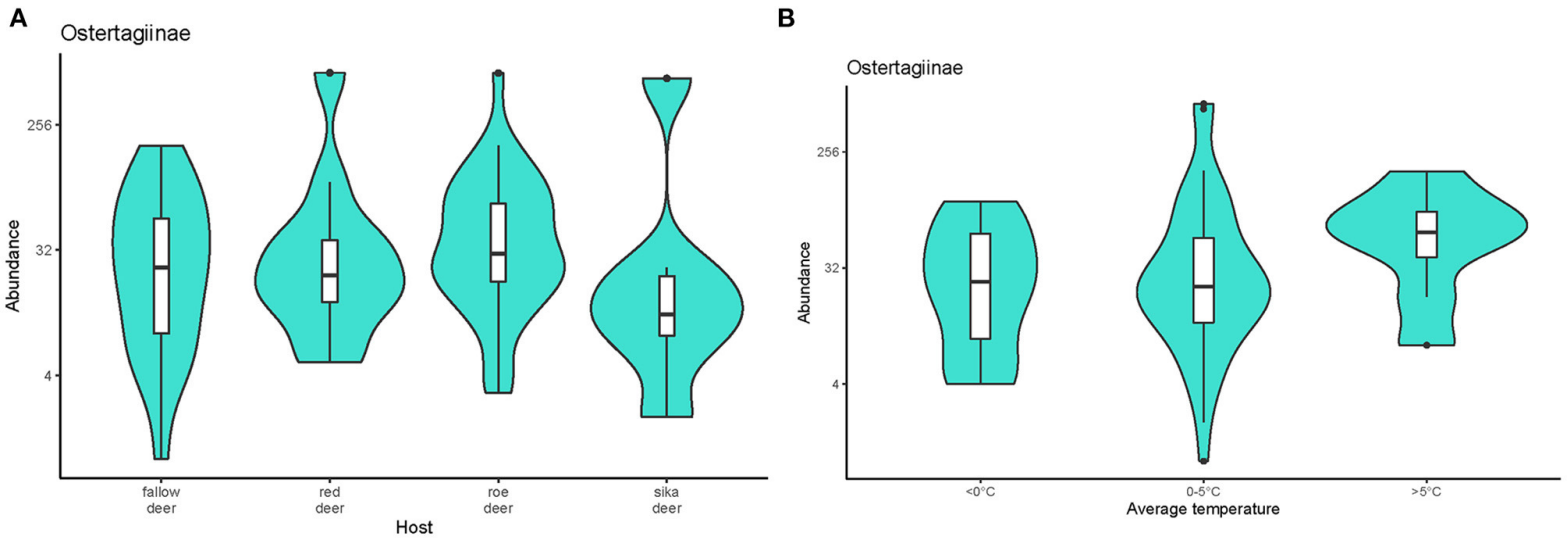
Violin plots representing the abundance of nematodes from the subfamily Ostertagiinae recovered **(A)** from the cervid hosts and **(B)** during periods with different monthly average temperatures. Medians are displayed in the boxplots inside the violin plots. Black dots symbolize outliers. The data on pooled ostertagid abundance were log-transformed.

The model selected based on AICc (Supplementary Table 1B) indicated that the abundance of ostertagids was positively affected by combined factors of host species and monthly average temperature (w = 0.447). Estimates from the selected model are given in the Supplementary Table 2. The abundance of these parasites was higher in roe deer than the other cervids (Figure 4A). Abundance was significantly lower in animals culled during months with average temperatures <0 °C than at warmer times of the year (Figure 4B). No effect of sex or age was detected.

## Discussion

Abomasal nematodes have been repeatedly identified as the most prevalent and abundant GI parasites in cervids (1, 51). Consistent with these results, 92% of the animals in our survey were infected with abomasal nematodes. Most of the abomasa were infected with one to three nematode species. Co-infections with multiple nematode species have been previously described (2, 16, 51). A study conducted in the CR in the 1970s listed *A. sidemi* as the dominant parasite of sika deer and reported this parasite in sympatric deer and mouflon (52). We found this parasite in a wide range of autochthonous hosts, including red deer, roe deer, fallow deer, and moose across the country. This expansion may be due to the rapid increase in the number of sika deer and its dynamic spread (53). The animals in our study were predominantly infected with *A. sidemi*, accompanied by several ostertagids. We report for the first time the presence of *S. houdemeri* in the Czech territory and in all species studied except moose and white-tailed deer. The occurence of this nematode was known in the past only in Muntjack deer (*Muntjacus munjack*) (54) and sambar (*Rusa unicolor*) (20) from Vietnam and in sika deer from Japan (55). It has been identified more recently in sika deer from Germany (56) and Austria (57), although this cervid species was acclimatized in Europe more than a 100 years ago. *Spiculopteragia houdemeri* was probably introduced into the CR after the fall of the Iron Curtain when the migration of infected sika deer from Austria was allowed (57). This parasite currently occurs in native cervids in localities co-habited with sika deer. Most of the nematode species detected in our survey infected all host species, except for *S. antipini* and *M. dagestanica*, which are considered typical parasites of moose (58), and *H. contortus*, which was found only in roe deer and white-tailed deer.

Both the richness of nematode species and the abundance of ostertagids were higher in the abomasa of roe deer compared to the other host species. Hofman (59) divided ruminants into three feeding types based on the morphophysiological differences in their digestive tracts and on their grazing behaviors: (i) concentrate selectors, (ii) browsers, and (iii) intermediate feeders. As a concentrate selector, roe deer have a different foraging behavior (including selective grazing of easily digestible plants) than the grazers such as mouflons or the other species studied, intermediate feeders with a dietary preference for grass. The defensive mechanisms against parasites also differ between these trophic groups. Grazers develop effective immune responses, but concentrate selectors tend to avoid infection (60). The avoidance of pastures used by other ruminants, and self-medication by foraging tannin-rich legumes, are behavioral methods that roe deer use to reduce the intake of infective larvae and thus nematode load (4, 61). These mechanisms, however, may be considerably limited in hunting grounds co-habited with a high density of more competitive cervid species and in fragmented agricultural landscapes. Interspecific competition for food sources can decrease body weight in roe deer fawns and induce stress (62–64) which may increase susceptibility to GI nematodes. The roe deer in our study harbored the typical nematodes *O. leptospicularis* and *S. spiculoptera*, similar to red deer. Unlike red deer, though, the roe deer were more frequently infected with *S. asymmetrica* and *S. houdemerii* in game reserves shared with fallow deer and sika deer, respectively. High intensities of infection with abomasal nematodes and the presence of atypical nematode species have been previously found in roe deer from an area inhabited by multiple species of cervids (38).

*Haemonchus contortus* contributed to the higher richness of abomasal nematodes in roe deer from hunting grounds where contact with livestock was possible. The transmission of *H. contortus* between roe deer and small domestic ruminants has previously been discussed (65). Co-infections with *H. contortus* and *A. sidemi* occur (24, 25), but only a few *H. contortus* individuals were detected in reported cases (8). We have not observed any co-occurrence of these nematode species in the male infrapopulations we investigated, but co-infections cannot be completely ruled out as we examined only subsample of infrapopulation. This parasite may not be able to compete with *A. sidemi* in the long term if its typical hosts, small domestic ruminants, are not present in hunting grounds. Similarly, *H. placei* infections gradually declined in sheep after reinfection with *H. contortus* on pasture where cattle were not present (66). The prevalence of *A. sidemi* was surprisingly high in locations where *H. contortus* was previously abundant (67). This high prevalence may have been caused by both the disappearance of potential sources of *H. contortus* during gradual changes from agricultural landscapes inhabited by livestock to forest in recent decades in the CR and by the dispersion of sika deer into this area (53, 68).

Only limited information is available on *A. sidemi* as part of the community of abomasal nematodes, indicating that this parasite is more abundant than native nematode species (25, 69). Similarly, most of the nematodes detected in our survey were *A. sidemi*. This parasite prevailed over the ostertagids in its prevalence, maximal numbers of recovered individuals, and biomass. The median abundance of *A. sidemi* did not differ significantly between host species, but fallow deer were more likely to be infected with higher numbers of *A. sidemi* than the other cervids. Management may have had a confounding effect on the intensity of *A. sidemi* infection, because the majority (almost 80%) of fallow deer were culled at game reserves with higher animal densities compared to open hunting grounds. The density of cervid hosts and both the intensity and abundance of parasites have previously been positively correlated (36). Aggregation during additional feeding can also cause social stress that may reduce immunity (70–72). The transmission of nematodes in confined spaces could therefore be amplified by an increased susceptibility of the host. The dominance of *A. sidemi* and low species richness in fallow deer could be due to different susceptibilities of nematode species to anthelmintic treatment (73). Fallow deer in the game reserves in this survey were dewormed annually at the beginning of the year (January 2018 and 2019).

The intensity of abomasal nematodes was highest in roe deer from the Židlov game reserve (50°37′14″ N., 14°51′37″ E), which is co-habited by the European bison. The European bison is highly susceptible to *A. sidemi* infection and infected individuals often carry thousands of nematodes (29, 32), so this host species is a significant source of infection for sympatric ruminants. Red deer and moose may play key roles in the introduction of *A. sidemi* into new localities due to their extensive home range and long-distance migration (74). The spread of *A. sidemi* with red deer from the CR to neighboring countries can be expected in the near future, similar to that of the giant liver fluke *Fascioloides magna* recently observed in Bavaria (German territory adjacent to the CR) (75). Intensities of infections with *A. sidemi* become high after it is introduced into hunting grounds, especially in roe deer, which may be highly susceptible. As the most abundant and widely distributed cervid in the CR (76), roe deer contribute significantly to the local spread of *A. sidemi* in the wild. Roe deer may also pose potential risks of transmitting *A. sidemi* to livestock, especially in fragmented agricultural landscapes that allow contact between wild and domestic ruminants. Susceptibility to this parasite has previously been confirmed in sheep (77) and cattle (78).

The abundance of *A. sidemi* was not significantly affected by season or temperature. The intensity of infection was highest in a roe deer culled in November 2016, probably due to the accumulation of nematodes by grazing during summer and early autumn. We found no support for an important role of arrested larval development in winter; the numbers of recovered larvae were negligible, similar to a previous study (32). The L5 juvenile stage, if present, made up a minority of the nematode infrapopulation, and we isolated from 1 to >2,000 adults. A low prevalence of arrested larvae and winter transmission of trichostrongylids may be more common in wild ruminants than livestock (4, 79).

Environmental conditions, especially temperature and humidity, contribute significantly to the probability of survival and transmission of parasites, with developmental cycles involving free-living stages (80). The abundance of ostertagids in our study varied with average temperature. The abundances of these nematodes were significantly higher in months with average temperatures >0°C than in colder months (December 2016, February 2018, and January 2019). Winter intensities of ostertagid infections were previously lower in cervids from areas with mild climates (37, 38), possibly due to a lower transmission during winter (35) and to a gradual decline of infrapopulations of this parasite in summer and autumn. Other factors such as the anthelmintic treatment of farmed cervids may also have contributed to the decline.

Based on our data, we cannot confirm the effect of acquired immunity on parasitic load for any group of abomasal nematodes, because nematode abundance did not differ between older animals and fawns. Some previous studies based on necropsies (11, 81) as well as the metabarcoding of larvae isolated from fecal samples (82) have also demonstrated a limited effect on the abundance and intensity of infection by ostertagids. The abundance of the parasites was also not affected by the sex of the host.

Our study was limited by specific factors, mainly the type of examination method used. Sample size generally depended on the culling plan of individual hunting grounds and on the willingness of their owners to provide biological material. Hunting laws in the CR allow the culling of ungulates only during a strictly defined period. We therefore could not monitor the seasonal dynamics of parasitic load throughout the year. We also could not obtain an equal representation of all groups, because the legal hunting season varies between species, sexes, and age cohorts. For example, obtaining females was not possible when the immunosuppressive effect of parturition on nematode infection could be expected. Loads with abomasal nematodes could be affected by other factors that could not be evaluated in this survey, such as different availabilities of nutrients or levels of supplementary feeding. The nematode abundances may also have been affected by the selection of culled animals based on their body condition. Despite these unavoidable limitations, our research provides realistic insights into the communities of abomasal nematodes in cervids under the current conditions in the CR.

## Conclusion

Cervids under the current climatic and environmental conditions of the CR were infected with a wide spectrum of abomasal nematode species, with the predominance of *A. sidemi*. This non-native parasite was detected in a wide range of hosts, whereas its occurrence and abundance were not significantly affected by either environmental (monthly average temperature) or host factors (species, age, sex). The population growth and spread of sika deer in addition to the diversity of autochthonous host species and their high sensitivity to *A sidemi* infections, have likely contributed to the recent invasive spread of this nematode amongst autochthonous ungulates. Our results demonstrated that *A. sidemi* can reach high intensities, especially in farmed fallow deer and in roe deer in hunting grounds. Red deer can introduce *A. sidemi* to new areas due to their distant migration, but roe deer can play a key role in the local spread of *A. sidemi* in the wild due to its widespread distribution and increasing numbers. Roe deer also pose a potential source of infection for livestock in sympatric zones. Longitudinal studies covering the summer and later spring are needed to obtain a comprehensive assessment of the seasonal dynamics of *A. sidemi* under the current climatic conditions of central Europe.

## Data Availability Statement

The original contributions presented in the study are included in the article/Supplementary Material, further inquiries can be directed to the corresponding author.

## Ethics Statement

The animal study was reviewed and approved by Institutional ethics and animal welfare committee of the Czech University of Life Sciences Prague.

## Author Contributions

JV and JM conceived and designed the study and interpretated the data. JV collected the samples. JM processed the samples (necropsies) and recovered and identified the parasites. JM and GB performed the statistical analyses and drafted the manuscript. JV and GB critically revised the manuscript. All authors accepted the final version of the manuscript.

## Funding

This research was financially supported by the Technology Agency of the Czech Republic, project No. TJ01000009.

## Conflict of Interest

The authors declare that the research was conducted in the absence of any commercial or financial relationships that could be construed as a potential conflict of interest.

## Publisher's Note

All claims expressed in this article are solely those of the authors and do not necessarily represent those of their affiliated organizations, or those of the publisher, the editors and the reviewers. Any product that may be evaluated in this article, or claim that may be made by its manufacturer, is not guaranteed or endorsed by the publisher.
