# Supplementary material for: Non-native Nematode Ashworthius sidemi Currently Dominates the Abomasal Parasite Community of Cervid Hosts in the Czech Republic
Source: Front Vet Sci. 2022 Apr 28;9:862092. doi: 10.3389/fvets.2022.862092 (PMC9096835; doi:10.3389/fvets.2022.862092)
Supplement: Supplementary file 1 [file Table_1.DOCX]

**Supplementary table 1:** Model selection for testing an influence of host and environmental factors on abundance of (A) *Ashworthius sidemi* and (B) nematodes from the subfamily Ostertagiinae. The selected models are in bold.

| **Model** | **Df** | **AIC** | **∆AIC** | **weight** |
| --- | --- | --- | --- | --- |
| **No effect** | **2** | **447.8** | **0.00** | **0.405** |
| Age | 3 | 479.2 | 1.33 | 0.208 |
| Temperature | 3 | 479.7 | 1.87 | 0.159 |
| Age+temperature | 4 | 481.0 | 3.19 | 0.082 |
| Species | 5 | 482.4 | 4.54 | 0.042 |
| Sex | 6 | 482.7 | 4.91 | 0.035 |
| Species+temperature | 6 | 483.4 | 5.63 | 0.024 |
| Age+species | 6 | 484.1 | 6.29 | 0.017 |
| Age+sex | 7 | 484.1 | 6.29 | 0.017 |
| Sex+temperature | 7 | 485.0 | 7.17 | 0.011 |

| **Species + temperature** | **6** | **396.3** | **0.00** | **0.447** |
| --- | --- | --- | --- | --- |
| Species + temperature + age | 7 | 398.4 | 2.10 | 0.157 |
| Species + temperature +sex | 8 | 399.5 | 3.20 | 0.090 |
| Temperature | 3 | 400.0 | 3.72 | 0.070 |
| Species | 5 | 400.1 | 3.75 | 0.069 |
| Age + temperature | 4 | 400.6 | 4.26 | 0.053 |
| Sex + temperature | 11 | 400.8 | 4.44 | 0.049 |
| Species | 9 | 401.9 | 5.54 | 0.028 |
| Species+sex+temperature | 6 | 402.3 | 5.95 | 0.023 |
| Age+temperature | 12 | 403.2 | 6.84 | 0.015 |
