# Supplementary material for: Non-native Nematode Ashworthius sidemi Currently Dominates the Abomasal Parasite Community of Cervid Hosts in the Czech Republic
Source: Front Vet Sci. 2022 Apr 28;9:862092. doi: 10.3389/fvets.2022.862092 (PMC9096835; doi:10.3389/fvets.2022.862092)
Supplement: Supplementary file 2 [file Table_2.DOCX]

**Supplementary table 2** Model estimates of the selected model (Supplementary table 1) testing the effect of host species (fallow deer as reference) and average temperature on abundance of nematodes from the subfamily Ostertagiinae.

| **Variable** | **Estimate** | **Std. error** | **t-value** | **p** |
| --- | --- | --- | --- | --- |
| Intercept | 1.93 | 0.31 | 6.21 | *** |
| Red deer | 0.34 | 0.52 | 0.66 |  |
| Roe deer | 1.18 | 0.40 | 2.99 | ** |
| Sika deer | 0.06 | 0.60 | 0.11 |  |
| Temperature | 0.11 | 0.046 | 2.43 | * |

Residual standard error: 1.656 on 96 degrees of freedom Multiple R-squared: 0.1668, Adjusted R-squared: 0.1321 F-statistic: 4.804 on 4 and 96 DF, p-value: 0.001416, ***: p<0.05, ****: p<0.01, ***: p<0.001
